# Global research progress on radiofrequency ablation in cardiology: A bibliometric analysis (2004–2023)

**DOI:** 10.1097/MD.0000000000038498

**Published:** 2024-06-07

**Authors:** Mei Liang, Jing Zhang, Guohui Li, Pengyu Wang

**Affiliations:** a Department of Cardiology, Yuxi People’s Hospital, Yuxi City, China; b Department of Emergency, First People’s Hospital of Yunnan Province, China; c Department of Cardiology, Zhongshan Hospital, Yuxi City, Yunnan Province, China.

**Keywords:** bibliometric, cardiology, global, hotspots, radiofrequency ablation

## Abstract

In recent years, significant advancements in radiofrequency ablation technology have notably enhanced arrhythmia treatment in cardiology. Technological advancements and increasing clinical adoption have made radiofrequency ablation a key therapy in improving life quality for patients with conditions like atrial fibrillation (AF). Consequently, there has been a marked increase in research output, underscoring the technology’s significance and its potential in cardiology. Aims to comprehensively analyze cardiology’s radiofrequency ablation research trends, identifying leading countries and institutions in international collaborations, key researchers’ contributions, and evolving research hotspots. The study, based on the Web of Science Core Collection database, reviewed the literatures from 2004 to 2023. CiteSpace 6.2.R7 Basic was used for bibliometric analysis, which examined annual publication trends, international collaboration networks, key authors, leading research institutions, major journals, keyword co-occurrence and clustering trends. Analyzing 3423 relevant articles, this study reveals a consistent growth in cardiology radiofrequency ablation research since 2004. The analysis shows that the United States, Germany, and France hold central roles in the international collaboration network, with leading authors from premier US and European institutions. Keyword cluster analysis identifies “atrial flutter” and “ventricular tachycardia” as current research focal points. Cardiology radiofrequency ablation research shows a growth trend, led by the United States and European countries. Research hotspots are concentrated on the diverse applications of radiofrequency ablation technology and the treatment of AF. Future studies may increasingly focus on technological innovation and the deepening of clinical applications.

## 1. Introduction

With the aging global population, the incidence of arrhythmias, especially atrial fibrillation (AF), is increasing globally.^[1–3]^ AF increases the risk of stroke and heart failure and is linked to higher mortality rates.^[3–5]^ A study^[2]^ reports that the number of AF patients in the United States is expected to reach 6 to 16 million by 2050, and at least 72 million in Asia, significantly burdening healthcare systems. Other arrhythmias, including ventricular tachycardia, atrial flutter, and preexcitation syndromes, also threaten patient health. The widespread and complex nature of arrhythmias highlights the need for effective treatment strategies.

Traditionally, arrhythmias were managed mainly through pharmacotherapy, which has limited effectiveness and potential side effects.^[6,7]^ Radiofrequency ablation technology is a significant innovation in arrhythmia treatment. It targets the heart tissue causing abnormal rhythms and delivers radiofrequency energy to specific areas, destroying or isolating the problematic tissue to restore normal rhythm.^[8]^ In treating AF, radiofrequency ablation commonly isolates pulmonary veins.^[9]^ It is effective in locating and eliminating abnormal circuits in ventricular tachycardia.^[10]^ Radiofrequency ablation has lower complication risks and reduces long-term medication dependence compared to pharmacotherapy. Thus, it represents a major advance in arrhythmia treatment and significantly impacts public health.

Radiofrequency ablation technology uses radiofrequency energy to destroy heart tissue that generates abnormal electrical signals, restoring normal heart rhythm.^[11]^ This method’s advantages are its precision, rapid recovery, and minimal patient trauma. With advances in medical technology and devices, radiofrequency ablation has evolved from experimental to a mature treatment, offering safer, more effective options for arrhythmia patients. Precise catheter positioning systems and advanced imaging technologies have greatly enhanced the safety and effectiveness of radiofrequency ablation.^[12,13]^ New ablation catheters and 3-dimensional electrophysiological navigation systems have improved treatment precision and safety, aiding in accurately locating and ablating abnormal rhythm sources. New ablation catheters and 3D electrophysiological navigation systems have enhanced treatment precision and safety, helping physicians accurately locate and ablate sources of abnormal rhythms.

Despite its significant achievements in cardiology, further research on radiofrequency ablation is needed to assess its effectiveness in various arrhythmias, long-term effects, and comparative efficacy. With ongoing research, more clinical and experimental studies on radiofrequency ablation are emerging, offering valuable insights and guiding future research directions. Consequently, comprehensively analyzing the past 2 decades’ research trends and applications of radiofrequency ablation technology is crucial. This analysis helps understand the technology’s evolution and effectiveness in cardiac disease treatment. Additionally, systematic literature review and analysis can pinpoint key technological and clinical milestones and future research avenues. This provides essential historical insights and future guidelines for medical professionals and researchers and informs patients about treatment options and outcomes.

## 2. Methods

### 2.1. Data sources and literature selection

This study used the Web of Science Core Collection database for literature retrieval, known for its comprehensiveness in interdisciplinary scientific citations and offering a wide range of high-quality, peer-reviewed literature. Covering fields like medicine, biology, engineering, and social sciences, it is an ideal source for analyzing trends in radiofrequency ablation applications in cardiology. To ensure comprehensive literature coverage, we used topic search (TS) strategies, including titles, abstracts, and keywords. Criteria for literature selection included: publications dated between January 2004 and December 2023; focus on radiofrequency ablation in cardiology; limitation to English-language; peer-reviewed “reviews” and “articles” only. Literature retrieval was completed on January 8, 2024. Search strategy details are in Table 1.

**Table 1:** Retrieval Strategy.

Search number	Search strategy (TS = topic search)	Number of literatures
#1	“Radiofrequency Ablation” OR “RF Ablation” OR “Thermal Ablation”	24,943
#2	“Cardiology” OR “Cardiac” OR “Heart Disease” OR “Arrhythmia” OR “Electrophysiology”	582,327
#3	#1 AND #2	3423

### 2.2 Analysis tools

The study used CiteSpace 6.2.R7 Basic, a JAVA application by Dr Chaomei Chen of Drexel University, for co-occurrence and co-citation analysis, visualizing scientific field trends and frontier areas. CiteSpace facilitated data import, node selection, parameter setting, and network graph creation, helping identify research hotspots, trends, and key nodes.

### 2.3. Data analysis

We exported complete records from the Web of Science Core Collection, including detailed article information and referenced citations. For processing ease, we exported the data in plain text format (TXT files) and imported them into CiteSpace. CiteSpace was used to create scientific knowledge maps, revealing both the structure of scientific knowledge and trends in radiofrequency ablation in cardiology.

The time-slicing technique was employed, setting the time range from January 2004 to December 2023, with each year as an independent unit. This method enabled detailed tracking and analysis of long-term trends and changing patterns over time. We focused on burst terms in the literature to identify emerging and declining themes in the field.

For in-depth analysis, centrality indices were calculated, quantifying a node’s importance based on its connections in the network. A higher centrality index indicates a node’s greater role in information dissemination and influence on research directions. Streamlined sentence for conciseness. Centrality indices were calculated based on co-citation relationships in CiteSpace.

A threshold of top n = 30 was set, focusing on the top 30 most significant items in each time slice. The Pathfinder network pruning algorithm was applied to enhance the network analysis’s clarity and usefulness. The burst detection algorithm by Goldberg et al^[14]^ was used to identify emerging themes, aiding in understanding recent developments and future research directions.

## 3. Results

### 3.1. Annual publication trend analysis

**Figure 1.**
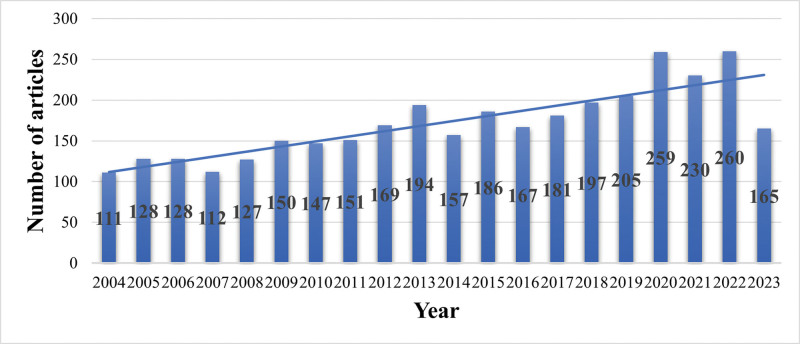
Publication trend in cardiology radiofrequency ablation (2004–2023). Each bar represents a year with the corresponding number of articles labeled at the middle of each bar.

From 2004 to 2023, a total of 3423 original articles met the search criteria, including 3002 articles and 421 review articles. The publication volume exhibited an overall upward trend (Fig. 1), marked by several growth points. Beginning with 111 articles in 2004, there was a steady increase, reaching a peak of 194 articles in 2013. This peak, twenty-five articles more than in 2012, signified a substantial increase in research interest. In 2014, the number slightly dropped to 157 articles but resumed growth, reaching 205 articles in 2019, indicating ongoing research activity. A significant surge to 259 articles in 2020, linked to global events or scientific discoveries, suggests increased research in this area. The publication volume decreased slightly to 230 in 2021, then rose to 260 in 2022, the highest in this period. Notably, there was a decline to 165 articles in 2023, potentially due to incomplete data collection or a shift in research focus. Overall, the data show sustained interest and increasing academic output in cardiology radiofrequency ablation over time. Despite recent fluctuations, the long-term trend indicates consistent attention and growing research efforts in this field.

### 3.2. Country/region analysis

**Figure 2.**
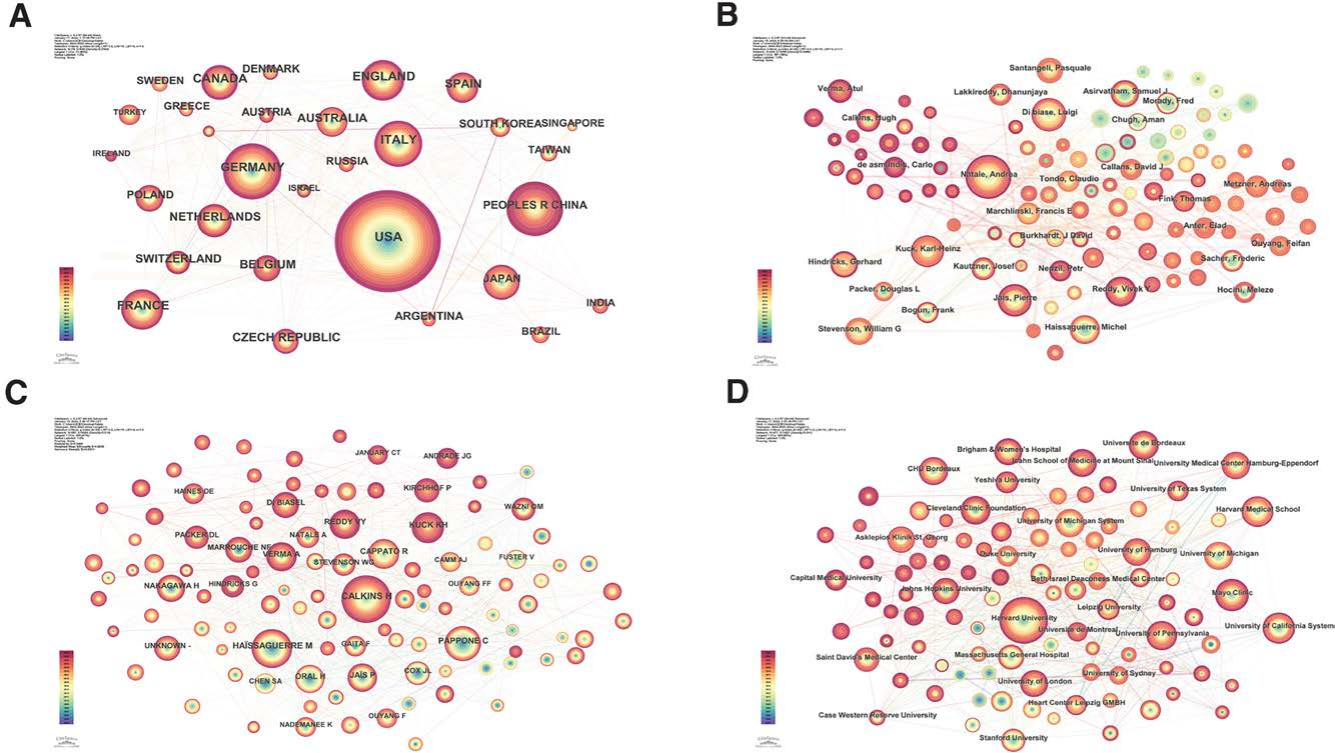
Comprehensive network analyses in cardiology radiofrequency ablation research. (A) Country/region contribution and collaboration map. (B) Author collaboration network. (C) Cited authors’ influence network. (D) Institutional collaboration network.

In the field of cardiology radiofrequency ablation research, we analyzed 3423 publications that involve at least seventy-eight countries/regions. As indicated in Table 2, the United States, Germany, and China are the top 3 contributors with 1307, 407, and 399 publications respectively. This data underscores their substantial contributions to the field. Furthermore, the country-based research network map (Fig. 2A) reveals a high-density collaboration pattern (n = 78, E = 650, density = 0.216), which illustrates extensive global cooperation.

**Table 2:** Top 10 countries/regions.

Rank	Country	Centrality	Count
1	USA	0.33	1307
2	Germany	0.15	407
3	Peoples R. China	0.01	399
4	Italy	0.11	293
5	England	0.17	227
6	France	0.08	224
7	Japan	0.01	180
8	Canada	0.10	166
9	Netherlands	0.02	165
10	Spain	0.06	143

Additionally, we employed centrality metrics to evaluate the influence and connectivity of countries within the international collaboration network. The United States, with a centrality of 0.33, is positioned at the core of this network, followed by the United Kingdom and Germany with centralities of 0.17 and 0.15, respectively. These centrality rankings highlight the pivotal roles these countries play in the network, facilitating significant collaborative links between various global research entities.

### 3.3. Author analysis

In the field of cardiology radiofrequency ablation, 834 authors contributed to publications, with the top ten listed in Table 3. Andrea Natale of Marche Polytechnic University, USA, led with 101 articles, demonstrating his substantial academic contribution and leadership. Luigi Di Biase from Albert Einstein College of Medicine, USA, followed with fifty-five publications, reflecting his notable research contribution. Pierre Jais from France’s Cleveland Clinic Foundation ranked third with fifty-two publications, displaying France’s active role in cardiac electrophysiology. Karl-Heinz Kuck from Schleswig Holstein University Hospital, Germany, and Gerhard Hindricks from Charite Universitatsmedizin Berlin, with 48 and 39 publications respectively, underscore Germany’s influence in this field. Vivek Y. Reddy from Icahn School of Medicine at Mount Sinai, USA, published forty-five articles, ranking fifth, highlighting his research contributions. This data indicates the USA and Europe’s leading roles in radiofrequency ablation research, emphasizing the field’s international and diverse nature.

**Table 3:** Top 10 authors.

Rank	Author	Country	Institution	Count
1	Natale, Andrea	USA	Marche Polytechnic University	101
2	Di biase, Luigi	USA	Albert Einstein College of Medicine	55
3	Jais, Pierre	France	Cleveland Clinic Foundation	52
4	Kuck, Karl-Heinz	Germany	Schleswig Holstein University Hospital	48
5	Reddy, Vivek Y.	USA	Icahn School of Medicine at Mount Sinai	45
6	Hindricks, Gerhard	Germany	Charite Universitatsmedizin Berlin	39
7	Haissaguerre, Michel	France	Fdn Bordeaux University	33
8	Stevenson, William G.	USA	Brigham & Womens Hosp	31
9	Asirvatham, Samuel J	USA	Mayo Clinic	31
10	Santangeli, Pasquale	USA	Cleveland Clinic Foundation	29

The author research network map (Fig. 2B) exhibited lower density (n = 834, E = 3099, density = 0.0089), suggesting some collaboration and communication, but not densely interconnected. This implies that while collaboration exists among prolific authors, there’s room to strengthen and expand the broader collaboration network.

Based on citation counts (Table 4), Hugh Calkins from Johns Hopkins University led with 891 citations in twenty-six articles. Michel Haissaguerre, representing Fdn Bordeaux University, had 607 citations in thirty-three articles. Notably, C. Pappone, with just 3 publications, achieved a high citation count of 491, averaging 163.67 citations per article. Karl-Heinz Kuck and Riccardo Cappato also showed significant research impact. The cited authors’ research network map (Fig. 2C) displayed a lower density (n = 987, E = 9265, density = 0.019). This relatively low density suggests that while the map includes numerous influential authors within the field of cardiology radiofrequency ablation, the connections between them, as measured by citation links, are sparse. This sparsity indicates that although these authors are frequently cited within the field, they do not necessarily collaborate directly with each other on research projects. The map highlights potential areas where increased collaboration among these influential researchers could significantly benefit the field.

**Table 4:** Top 10 authors with the highest citations.

Rank	Author	Country	Institution	Count	Total citations	Average citations
1	Calkins, Hugh	USA	Johns Hopkins University	26	891	34.27
2	Haissaguerre, Michel	France	Fdn Bordeaux University	33	607	18.39
3	Pappone, C.	USA	San Raffaele Univ Hosp	3	491	163.67
4	Kuck, Karl-Heinz	Germany	Schleswig Holstein University Hospital	48	405	8.44
5	Cappato, Riccardo	Italy	IRCCS Humanitas Res Ctr	2	367	183.5
6	Reddy, Vivek Y	USA	Icahn School of Medicine at Mount Sinai	45	363	8.07
7	Verma, Atul	Canada	McGill University	23	351	15.26
8	Jais, Pierre	France	Cleveland Clinic Foundation	52	327	6.29
9	Oral, Hakan	USA	University of Michigan	9	325	36.11
10	Nakagawa, Hiroshi	USA	Columbia University	17	293	17.24

### 3.4. Institutional analysis

Harvard University, with a centrality score of 0.05 and 193 publications, leads in radiofrequency ablation research in cardiology, as shown in Table 5, highlighting its dominant role and prolific output in this domain. Mayo Clinic follows with a centrality of 0.03 and 98 publications, indicating its active involvement and substantial impact in the field. The University of California System and Harvard Medical School, with publication counts of 95 and 93 and centralities of 0.04 and 0.01 respectively, underscore their significance in this research area. The University of Pennsylvania and Brigham & Women’s Hospital, each with a centrality of 0.06 and 89 and 84 publications respectively, demonstrate their pivotal research roles and high publication output. Despite a lower publication count of seventy-nine, Universite de Bordeaux stands out with the highest centrality of 0.07, indicating its critical role in global collaboration and research networks. Cleveland Clinic Foundation and CHU Bordeaux, with centralities of 0.06 and 0.08 and publication counts of 78 and 77, respectively, underscore the prominent role of France in this field. The institutional research network map (Fig. 2D) reveals a low density (n = 477, E = 3521, density = 0.031), suggesting a need for increased collaboration and institutional synergy.

**Table 5:** Top 10 institutions.

Rank	Institution	Centrality	Count
1	Harvard University	0.05	193
2	Mayo Clinic	0.03	98
3	University Of California System	0.04	95
4	Harvard Medical School	0.01	93
5	University Of Pennsylvania	0.06	89
6	Brigham & Women’s Hospital	0.06	84
7	Universite de Bordeaux	0.07	79
8	Cleveland Clinic Foundation	0.06	78
9	CHU Bordeaux	0.08	77
10	University of Michigan	0.03	77

### 3.5. Journal analysis and most-cited articles

In radiofrequency ablation cardiology research (Table 6), the Journal of Cardiovascular Electrophysiology leads with 309 articles, representing 9.03% of the total, indicative of its extensive contributions to the field. Europace ranks second with 219 articles and 1769 citations, markedly surpassing other journals, emphasizing its influential and respected status in this domain. The Journal of Interventional Cardiac Electrophysiology, with 215 articles (6.28%), ranks third, indicating potential for greater academic impact and recognition.

**Table 6:** Top 10 journals.

Rank	Journal	Count	Percent	IF2023
1	Journal of Cardiovascular Electrophysiology	309	9.03%	1.94
2	Europace	219	6.40%	5.93
3	Journal of Interventional Cardiac Electrophysiology	215	6.28%	1.70
4	Heart Rhythm	200	5.84%	4.10
5	Pace-Pacing and Clinical Electrophysiology	196	5.73%	1.74
6	Circulation-Arrhythmia and Electrophysiology	149	4.35%	6.34
7	JACC-Clinical Electrophysiology	106	3.10%	5.96
8	Journal of the American College of Cardiology	82	2.40%	14.80
9	International Journal of Cardiology	60	1.75%	2.51
10	Frontiers in Cardiovascular Medicine	54	1.58%	2.38
10	Circulation	54	1.58%	25.96

Note: IF (Impact Factor) statistics are as of December 27, 2023.

The top ten most-cited articles in cardiology radiofrequency ablation (Table 7) clearly highlight the central themes and key contributors in the field. Karl-Heinz Kuck’s study “Cryoballoon or Radiofrequency Ablation for Paroxysmal AF,” leading with 152 citations, underscores its notable impact on AF treatment. Hugh Calkins’ consensus statement articles on AF ablation, published in 2019, 2012, and 2017, each garnering over one thousand citations, highlight his leading role in the field. Research by Riccardo Cappato and Atul Verma also features in this list, reinforcing the crucial role of these highly-cited studies in both scientific and clinical aspects of cardiology radiofrequency ablation. Citations for these articles vary from 66 to 152, demonstrating their authoritative influence and guiding role in the global cardiology community.

**Table 7:** Top 10 titles.

Rank	Title	First author	Journal	Publication year		Total citations	DOI
1	Cryoballoon or Radiofrequency Ablation for Paroxysmal Atrial Fibrillation	Karl-Heinz Kuck	New England Journal of Medicine	2016	152		10.1056/NEJMoa1602014
2	2017 HRS/EHRA/ECAS/APHRS/SOLAECE expert consensus statement on catheter and surgical ablation of atrial fibrillation	Hugh Calkins	Europace	2019	112		10.1093/europace/eux274
3	2012 HRS/EHRA/ECAS expert consensus statement on catheter and surgical ablation of atrial fibrillation: recommendations for patient selection, procedural techniques, patient management and follow-up, definitions, endpoints, and research trial design	Hugh Calkins	Heart Rhythm	2012	94		10.1016/j.hrthm.2011.12.016
4	Updated worldwide survey on the methods, efficacy, and safety of catheter ablation for human atrial fibrillation	Riccardo Cappato	Circ Arrhythm Electrophysiol	2010	87		10.1161/CIRCEP.109.859116
5	2016 ESC Guidelines for the management of atrial fibrillation developed in collaboration with EACTS	Paulus Kirchhof	Europace	2016	80		10.5603/KP.2016.0172
6	2017 HRS/EHRA/ECAS/APHRS/SOLAECE expert consensus statement on catheter and surgical ablation of atrial fibrillation	Hugh Calkins	Heart Rhythm	2017	79		10.1016/j.hrthm.2017.05.012
7	Catheter Ablation for Atrial Fibrillation with Heart Failure	Nassir F Marrouche	New England Journal of Medicine	2018	750		10.1056/NEJMoa1707855
8	Approaches to catheter ablation for persistent atrial fibrillation	Atul Verma	New England Journal of Medicine	2015	720		10.1056/NEJMoa1408288
9	2012 HRS/EHRA/ECAS Expert Consensus Statement on Catheter and Surgical Ablation of Atrial Fibrillation: recommendations for patient selection, procedural techniques, patient management and follow-up, definitions, endpoints, and research trial design	Hugh Calkins	Europace	2012	670		10.1093/europace/eus027
10	HRS/EHRA/ECAS expert Consensus Statement on catheter and surgical ablation of atrial fibrillation: recommendations for personnel, policy, procedures and follow-up. A report of the Heart Rhythm Society (HRS) Task Force on catheter and surgical ablation of atrial fibrillation	Hugh Calkins	Heart Rhythm	2007	660		10.1016/j.hrthm.2007.04.005

### 3.6. Research hotspots and co-occurrence keyword analysis

**Figure 3.**
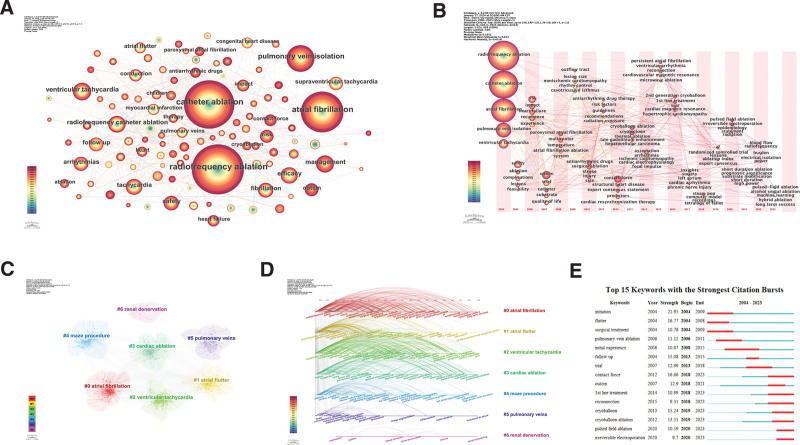
Keyword and theme analysis in cardiology radiofrequency ablation research. (A) Keyword co-occurrence map. (B) Keyword time zone view. (C) Keyword cluster view. (D) Timeline view of keyword clusters. (E) Keyword burst view.

An analysis of 3423 article titles and abstracts yielded a keyword co-occurrence map with 709 nodes and 7681 links (Fig. 2D). Table 8 lists the top ten most frequent keywords, with “radiofrequency ablation” leading with 1893 mentions but a centrality of 0.00. This suggests its prevalent mention, yet a low centrality in the research network, due to its broad application in diverse research contexts. “Catheter ablation” (1726 occurrences) and “AF” (1297 occurrences) follow in frequency. “Ventricular tachycardia” and “management,” each with a centrality of 0.04, lead among the top ten central keywords, indicating their prominence as research topics in the field. In centrality, “follow up” (0.06 centrality, 212 occurrences) tops the list, followed by “lesions” (0.05 centrality, eighty-seven occurrences), highlighting these as emerging focal points in the research network. This trend underscores the evolving research emphasis and dynamic nature of the cardiac electrophysiology field. CiteSpace created a keyword timezone view to visually depict the temporal emergence of keywords, effectively illustrating the evolution of high-frequency keywords (Fig. 3A),and these keywords were presented in a timezone view (Fig. 3B).

**Table 8:** Top 10 keywords.

Rank	Keyword	Centrality	Count
1	Radiofrequency ablation	0.00	1893
2	Catheter ablation	0.00	1726
3	Atrial fibrillation	0.01	1297
4	Pulmonary vein isolation	0.02	571
5	Ventricular tachycardia	0.04	314
6	Management	0.04	298
7	Efficacy	0.01	260
8	Radiofrequency catheter ablation	0.03	248
9	Arrhythmias	0.02	228
10	Tachycardia	0.02	225

### 3.7. Keyword cluster analysis

The log-likelihood ratio algorithm aggregated 709 keywords into 7 clusters (Fig. 3C). The average silhouette values of clusters 0 to 6, at 0.741, exceed 0.7, indicating strong homogeneity. Each cluster was characterized by index words derived from the keywords. The largest cluster, labeled “AF” (#0), reflects its prominence as a key research theme in cardiology radiofrequency ablation. The second-largest cluster, “atrial flutter” (#1), and the third-largest, “ventricular tachycardia” (#2), reveal diverse applications and research directions in arrhythmia treatment using radiofrequency ablation. The other clusters are labeled “cardiac ablation,” “maze procedure,” “autonomic nervous system,” and “renal denervation,” highlighting the varied applications of radiofrequency ablation in cardiology, encompassing cardiac surgery, neuromodulation, and hypertension treatment. To facilitate intuitive comprehension, these clusters were presented in a timeline view (Fig. 3D).

### 3.8. Burst analysis

CiteSpace was employed to identify the most prominent keyword bursts in cardiology radiofrequency ablation research from 2002 to 2022 (Fig. 3E). The top fifteen keywords exhibiting the highest burst strengths represent a marked increase in citation frequency over specific periods, signaling emerging trends and developments in the field. The keyword “initiation” demonstrated a citation burst from 2004 to 2009, with a strength of 21.86, indicating a phase of intensive early research in radiofrequency ablation. Concurrently, “flutter” and “supraventricular tachycardia” exhibited citation bursts with strengths of 16.71 and 13.06, respectively, reflecting significant research advancements in these arrhythmic conditions. “Pulmonary vein ablation” experienced a notable citation burst from 2006 to 2011, correlating with the increasing emphasis on pulmonary vein isolation for AF treatment. The keywords “cryoballoon” and “cryoballoon ablation” initiated their citation bursts in 2019, persisting through 2023, indicating the growing relevance of this novel technology. Recently, “pulsed field ablation” (PFA) has shown a citation burst starting in 2020, with a strength of 10.58, signaling its emergence as a rapidly evolving and recognized new area in the field.

## 4. Discussion

This study conducts a comprehensive literature analysis on cardiology radiofrequency ablation from 2004 to 2023, aiming to uncover the field’s research trends and developments. Employing bibliometric methods and CiteSpace, this study systematically evaluates radiofrequency ablation technology’s application trends, key research areas, and future potential. The analysis captures not only the temporal dynamics of research activities but also illuminates the contributions of leading countries, institutions, and authors, alongside the evolution of keywords in the field. This provides a holistic view of the development in cardiology radiofrequency ablation and suggests potential avenues for future research.

This study represents the first comprehensive bibliometric analysis of developmental trends in cardiology radiofrequency ablation. Between 2004 and 2023, there was a consistent upward trend in publications in this field, indicating sustained scientific interest and rising clinical treatment demands. This trend correlates with the maturation and extensive application of radiofrequency ablation technology, particularly in treating AF. Notable spikes in publication volume, specifically in years like 2013 and 2020, drew our attention. These spikes might be attributable to key scientific discoveries, technological advancements, or the dissemination of significant clinical trial findings in the field. For example, the 2013 revision of AF treatment guidelines by major cardiology societies,^[15]^ and the 2020 ESC guidelines for AF diagnosis and treatment,^[16]^ likely spurred research in this area. The 2020 literature surge could be linked to global health events,^[17]^ with an increase in cardiovascular disease mortality, notably affecting Black, Hispanic, and Asian populations. These events spurred the research community to delve deeper into cardiology treatment technologies. Additionally, we observed shifts in research focus, particularly towards emerging treatments like cryoballoon ablation (CB-A) and PFA, potentially contributing to publication volume fluctuations.

The distribution of contributions and author influence across countries illuminates the global research landscape’s characteristics. The United States’ leading position in this field is evidenced by its high centrality and publication volume. This leadership is due to substantial investment in medical research and technological innovation, coupled with a concentration of premier medical research institutions. The prolific output of leading USA researchers, including Natale, Andrea, and Di Biase, Luigi, reflects not only individual achievements but also the USA’s predominant role in global cardiac electrophysiology research. Germany and France’s significant standing in this field mirrors their robust research capabilities and profound influence in cardiology. Contributions from Kuck, Karl-Heinz, and Haissaguerre, Michel emphasize their expertise in innovative treatment methods and clinical application research. This prominence may be attributed to the high emphasis on medical research and considerable funding support in European countries. While lower in centrality, participation from China and Japan exhibits rapid growth and active research endeavors in Asia, due to recent increases in scientific research and medical technology investments. Additionally, analyzing highly cited authors offers another lens through which to view the global research landscape. For example, the high citation frequency of Calkins, Hugh, and Pappone, C. demonstrates the international recognition and impact of their work, owing to its innovative nature, advanced techniques, and clinical applications.

The centrality and publication frequency of various institutions highlight their status and influence within the global research network. Harvard University and Mayo Clinic’s high centrality and publication volume signify their roles as major research hubs and as key drivers in the clinical application and research advancement of radiofrequency ablation technology. Their leading positions are due to advantages in research resources, expertise, and funding. The prominence of Harvard Medical School and the University of Pennsylvania underscores the leading role of top USA medical research institutions in cardiology radiofrequency ablation. These institutions are known for their strong research teams and extensive clinical experience, contributing to their leadership in producing high-quality research. Universite de Bordeaux and CHU Bordeaux’s high centrality demonstrates France’s significant influence in this field, indicative of its expertise in cardiovascular disease research and active participation in international collaboration. Furthermore, the performance of institutions such as Cleveland Clinic Foundation and the University of Michigan underscores their significance in research and innovation in radiofrequency ablation technology, due to their strengths in clinical trials, research infrastructure, and interdisciplinary collaboration. The centrality and frequency of these institutions not only indicate their ability to generate high-impact research but also suggest their role in establishing and sustaining broad research collaboration networks.

The distribution of journals and their citation patterns illuminate the characteristics of academic communication and research influence in this field. The publication volumes and citation count of journals not only mirror their academic standing but also indicate shifts in research focus and quality within the field. The Journal of Cardiovascular Electrophysiology, having the highest publication volume, signifies its prominence in the radiofrequency ablation technology field. Despite a lower impact factor, its high publication volume may reflect the journal’s comprehensive and inclusive coverage of radiofrequency ablation research. Conversely, the high citation counts of Europace and Heart Rhythm, especially Heart Rhythm’s high impact factor and citations, highlight their authoritative status and research quality in the field. This is due to the quality, innovativeness, and clinical impact of the articles published in these journals. Although The Journal of Interventional Cardiac Electrophysiology ranks high in publication volume, its low citation count suggests a need for improvement in academic recognition or impact. This disparity may stem from the journal’s specialized focus, its reader demographics, or the relevance of its article topics. Notably, Circulation, despite a lower publication volume, exhibits a leading position and significant academic influence in cardiology, evidenced by its extremely high impact factor and citation counts. This implies that in this field, research of high quality tends to attract more attention and recognition.

The substantial impact of highly cited articles illuminates key developments and turning points in this field. In the realm of AF treatment research, Karl-Heinz Kuck’s “Cryoballoon or Radiofrequency Ablation for Paroxysmal AF”^[18]^ and several consensus statements by Hugh Calkins^[19–23]^ have become pivotal in the literature. Their high citation counts not only signify a profound impact on clinical treatment practices but also reflect the widespread acceptance and popularity of these techniques in AF treatment strategies. Kuck’s study, comparing cryoballoon and radiofrequency ablation in AF treatment, provided essential information for clinical decision-making, contributing to its global citation prominence. Calkins’ papers, notably the 2019 and 2012 consensus statements, provide comprehensive guidelines on radiofrequency ablation for AF, addressing patient selection, techniques, and management, and have become foundational in the field. The widespread citation of these articles underscores their academic and clinical recognition and highlights the advancements and innovations in radiofrequency ablation technology for AF treatment.

Keyword co-occurrence analysis discloses the primary research focuses and trends in the field. The high occurrence of terms such as “radiofrequency ablation,” “catheter ablation,” and “AF” underscores the central role and extensive application of radiofrequency ablation technology in AF treatment. The frequent mention of these terms reflects the sustained importance of radiofrequency ablation in cardiac electrophysiology research and its ongoing development and innovation in AF treatment. The notably high frequency of “pulmonary vein isolation” and “ventricular tachycardia” underscores the focused research interest in these specific arrhythmic treatment methods, reflecting the acknowledged effectiveness of radiofrequency ablation in treating these conditions. The presence of keywords like “management” and “efficacy” highlights a crucial research direction, encompassing both the development of radiofrequency ablation technology and its management and effectiveness evaluation in clinical practice. Keyword cluster analysis further illuminates the field’s diverse research trajectories. The largest cluster, labeled “AF,” indicates this condition as the primary research focus. Other clusters, such as “atrial flutter” and “ventricular tachycardia,” showcase the application of radiofrequency ablation in various arrhythmic treatments. The emergence and labeling of these clusters highlight the diversity in radiofrequency ablation research, encompassing topics from basic science to clinical applications.

Burst analysis uncovers historical shifts in research focus and technological advancements. The 2004 citation burst of “initiation” may relate to the early application of radiofrequency ablation technology in cardiac disease treatment, with initial studies^[24–26]^ exploring how radiofrequency energy induces reparative changes in cardiac tissue to correct arrhythmic circuits. These initial studies established the foundation for using radiofrequency ablation in treating various arrhythmias, particularly AF. Since 2006, the increased citations of “pulmonary vein ablation” highlight the rising significance of pulmonary vein isolation in AF treatment. This strategy, by isolating atrial areas around the pulmonary veins that trigger fibrillation, effectively reduces AF episodes, establishing it as a standard treatment option.^[27,28]^ Since 2013, the sharp increase in citations for “cryoballoon” and “cryoballoon ablation” reflects the rapid development and acceptance of this technology in AF treatment. Cryoballoon technology, preferred over traditional radiofrequency ablation, is known for its consistent, predictable outcomes and reduced complication risks. Long-term clinical studies^[29,30]^ have confirmed the safety and efficacy of cryoballoon technology, demonstrating high success rates over 5 years and indicating its growing importance as a treatment option in AF therapy. The emergence of “PFA” by 2020 suggests the potential impact of this innovative technology. PFA, utilizing nonthermal electric field effects from microsecond pulses, effectively ablates cardiac tissue while maintaining structural integrity.^[8]^ The introduction of PFA may represent a novel direction in advancing radiofrequency ablation technology, with early clinical studies^[31,32]^ suggesting its potential as a cardiac ablation tool. Overall, burst analysis serves as a tool to identify and evaluate the research and technological dynamics in cardiology radiofrequency ablation. Each keyword citation burst signifies new research interests, treatment innovations, or updates in guidelines. These trends demonstrate past progress and guide future research directions, reflecting the field’s ongoing development and evolution.

However, the study has several limitations. First, it relies on data from the Web of Science Core Collection database. Although this database is extensive, it does not include all publications, especially those from smaller, non-indexed journals, which could lead to the omission of relevant studies. Second, the analysis is limited to articles published in English, introducing a language bias that may overlook significant research published in other languages. Additionally, the use of specific search terms to define the scope of article retrieval, while enabling precise retrieval of literature within a specific field, may also exclude some marginal but relevant studies.

Despite these limitations, the insights gained from our analysis underscore the significant strides made in the development of radiofrequency ablation technology over the past 2 decades. Radiofrequency ablation technology has seen significant development, establishing its importance in treating arrhythmias like AF and atrial flutter. Additionally, radiofrequency ablation has demonstrated significant potential in uncovering the deeper mechanisms of cardiac electrophysiology. The advent of technologies such as CB-A and PFA represents a shift towards a wider exploration of nonthermal ablation methods, setting the stage for future technological advancements. Furthermore, the growing emphasis on management and efficacy evaluation of treatment outcomes reflects the medical community’s ongoing efforts to optimize long-term therapeutic results. Interdisciplinary collaboration, particularly with neurology and endocrinology, underscores the potential of radiofrequency ablation technology in offering comprehensive treatment strategies. With continuous advancements in new technologies and the optimization of treatment strategies, radiofrequency ablation technology promises increasingly effective and safer options for treating cardiac diseases.

## Author contributions

**Conceptualization:** Pengyu Wang.

**Data curation:** Jing Zhang.

**Methodology:** Guohui Li.

**Supervision:** Pengyu Wang.

**Validation:** Mei Liang.

**Writing – original draft:** Mei Liang.

**Writing – review & editing:** Jing Zhang, Guohui Li.
